# Anaerobic metabolism induces greater total energy expenditure during exercise with blood flow restriction

**DOI:** 10.1371/journal.pone.0194776

**Published:** 2018-03-29

**Authors:** Miguel S. Conceição, Arthur F. Gáspari, Ana P. B. Ramkrapes, Edson M. M. Junior, Romulo Bertuzzi, Cláudia R. Cavaglieri, Mara Patrícia T. Chacon-Mikahil

**Affiliations:** 1 Exercise Physiology Lab., School of Physical Education, University of Campinas–Campinas/Brazil; 2 School of Physical Education and Sport, University of São Paulo, São Paulo, Brazil; 3 Endurance Sports Research Group (GEDAE-USP), School of Physical Education and Sport, University of São Paulo—São Paulo/Brazil; University of Tennessee Health Science Center College of Graduate Health Sciences, UNITED STATES

## Abstract

**Purpose:**

We investigated the energy system contributions and total energy expenditure during low intensity endurance exercise associated with blood flow restriction (LIE-BFR) and without blood flow restriction (LIE).

**Methods:**

Twelve males participated in a contra-balanced, cross-over design in which subjects completed a bout of low-intensity endurance exercise (30min cycling at 40% of $\dot{V}O_{2\max}$) with or without BFR, separated by at least 72 hours of recovery. Blood lactate accumulation and oxygen uptake during and after exercise were used to estimate the anaerobic lactic metabolism, aerobic metabolism, and anaerobic alactic metabolism contributions, respectively.

**Results:**

There were significant increases in the anaerobic lactic metabolism (P = 0.008), aerobic metabolism (P = 0.020), and total energy expenditure (P = 0.008) in the LIE-BFR. No significant differences between conditions for the anaerobic alactic metabolism were found (P = 0.582). Plasma lactate concentration was significantly higher in the LIE-BFR at 15min and peak post-exercise (all P≤0.008). Heart rate was significantly higher in the LIE-BFR at 10, 15, 20, 25, and 30min during exercise, and 5, 10, and 15min after exercise (all P≤0.03). Ventilation was significantly higher in the LIE-BFR at 10, 15, and 20min during exercise (all P≤0.003).

**Conclusion:**

Low-intensity endurance exercise performed with blood flow restriction increases the anaerobic lactic and aerobic metabolisms, total energy expenditure, and cardiorespiratory responses.

## Introduction

It is well known that moderate to high intensity endurance training (i.e., 60–90% of maximum oxygen uptake—$\dot{V}O_{2\max}$) is the principal exercise protocol to induce an increase in $\dot{V}O_{2\max}$ [1, 2]. However, a novel endurance exercise protocol, using lower limb blood flow restriction (BFR) during low-intensity (~40% of $\dot{V}O_{2\max}$) endurance exercise (LIE-BFR), has also been shown to significantly improve $\dot{V}O_{2\max}$ (6.4%) [3, 4], suggesting that performing endurance training at low intensity, when associated with blood flow restriction, can induce cardiorespiratory fitness, providing a great advantage. For instance, Abe and co-workers [3] reported increased $\dot{V}O_{2\max}$ following 24 training sessions of low-intensity cycle exercise (15min at 40% $\dot{V}O_{2\max}$) performed with BFR compared to the same exercise intensity without BFR. These findings suggested that peripheral perturbation induced by BFR in arterial and venous leg blood flow, including local hypoxia [5] and reduce venous return [6], produces elevated metabolic demand. Accordingly, the BFR may be related to an enhanced anaerobic metabolism during muscle contraction, resulting in metabolite accumulation, and thus stimulating cardiorespiratory (i.e., via metaboreflex and reduced venous return) [7] and muscular mechanisms [8] to possibly increase $\dot{V}O_{2\max}$.

Accordingly, some studies have suggested that during exercise performed under local hypoxia the aerobic metabolism is decreased, while the anaerobic metabolism (i.e., alactic and lactic energy systems) is increased [9, 10]. It is believed that increased anaerobic energy production may evoke higher metaboreflex activity and, consequently enhance heart rate (HR) response, cardiac output, and ventilation (${\dot{V}}_{E}$) to adequately supply O_2_ to exercising muscle and remove the metabolites generated from the anaerobic metabolism [11–13]. As a result, this compensatory mechanism produced by local hypoxia, together with a reduced venous return characteristic of BFR training, may overload central components of the cardiorespiratory system and lead to improvement in $\dot{V}O_{2\max}$ [14, 15]. Furthermore, Egan et al. [8] showed that higher blood lactate ([La^-^]_net_) accumulation, indicating greater contribution of the anaerobic metabolism, can result in significantly higher metabolic stress and consequently enhance mitochondrial biogenesis synthesis, a local factor which can also induce greater $\dot{V}O_{2\max}$. Although these are plausible hypotheses; both present the anaerobic metabolism as a trigger factor that still needs to be identified during LIE-BFR.

Therefore, the aim of the present study was to compare the energy system contributions (i.e., aerobic, anaerobic alactic, and lactic metabolisms) and total energy expenditure following low intensity endurance exercise associated with blood flow restriction (LIE-BFR) and low intensity endurance exercise without BFR (LIE). We hypothesized that LIE-BFR would increase energy production by the anaerobic lactic metabolism due to reduction in O_2_ delivery to thigh muscles induced by cuff pressure, and thus increase total energy expenditure.

## Methods

### Subjects

Twelve healthy sedentary male subjects voluntarily participated in this study (subject characteristics are described in Table 1). Participants were recruited via fliers and posters at the University. As inclusion criteria, participants should be young (18 to 30 years old) men, sedentary at most, without contraindications to the practice of bicycle exercise and with availability of schedule to comply with all visits of the project. As exclusion criteria, participants could not have any cardiovascular or metabolic disease or musculoskeletal injuries in the lower limbs that compromise their ability to perform the exercise protocol. In addition, no participant could have been engaged in resistance and/or endurance training at least six months prior to the study and they were advised to do not consume alcohol and drugs or any kind of artificial substance (e.g., supplements) at least one week before the study commencement. At last, participants were instructed to refrain from consuming caffeine and other energetic substances in the 24 hours preceding all visits. The experimental procedures and possible risks associated with the study were explained to all subjects, who provided written consent prior to participation. The study was approved by the Ethics Committee of the University of Campinas (process number: 848.145) and conducted in accordance with the policy statement regarding the use of human subjects conforming to the latest revision of the *Declaration of Helsinki*.

**Table 1:** Characteristics of the participants.

	n = 12
Age (years)	24.5 ± 4.0
Body mass (kg)	82.8 ± 12.6
Height (m)	1.80 ± 0.05
$\dot{V}O_{2\max}$ (mL/kg/min)	33.4 ± 4.6
PO_max_ (W)	222.3 ± 47.6
RCP (mL/kg/min)	26.6 ± 4.7
RCP ($\%\dot{V}O_{2\max}$)	79.7 ± 9.8
RCP (W)	182 ± 51.8
VT (mL/kg/min)	20.4 ± 4.3
VT ($\%\dot{V}O_{2\max}$)	61.1 ± 10.4
VT (W)	127.9 ± 53

Values are presented as mean ± SD. $\dot{V}O_{2\max}$: maximum oxygen uptake; PO_max_: maximal power output; RCP: respiratory compensation point; VT, ventilatory threshold.

### Experimental design

The study employed a counter-balanced, cross-over design in which all subjects completed a session of low intensity endurance exercise associated with blood flow restriction (LIE-BFR) and one session of low intensity endurance exercise (LIE) without BFR on the cycle ergometer. Prior to the first exercise session, participants underwent $\dot{V}O_{2\max}$ testing and one exercise familiarization session. Exercise trials were separated by a period of between 72 hours and one-week, during which subjects were instructed to maintain their habitual diet and physical activity patterns.

#### Cardiorespiratory testing ($\dot{V}O_{2\max}$)

Participants performed a maximum graded exercise test on a cycle ergometer with electromagnetic braking (Quinton model: Corival 400, Lode BV, Groningen, Netherlands). After resting on the bike for 5 min to establish cardiorespiratory parameters, participants commenced the incremental test protocol. Briefly, subjects commenced cycling at an initial load of 50 W for 1min and the workload was increased by 15 W/min until a workload of 200 W was reached, after which further increases were in 10 W/min increments until voluntary fatigue [3, 16]. Participants were instructed to maintain pedal frequency between 60 to 70 rpm. Gas exchange and HR data were collected continuously using an automated breath-by-breath metabolic system (CPX, Medical Graphics, St. Paul, Minnesota, USA) and HR transmitter (Polar Electro Oy, Kempele, Finland) connected to the gas analyzer. A test was considered maximal when two or more of the following criteria were met: a plateau in oxygen uptake, a respiratory exchange ratio greater than 1.1, heart rate >90% of the predicted maximal heart rate (i.e., 220-age), and a score > 17 in 6–20 perceived exertion scale [17, 18]. The maximum oxygen uptake was defined as the mean oxygen consumption values ($\dot{V}O_{2\max}$) over the final 30 s of the test. Ventilatory threshold (VT) was determined at the point of a non-linear increase in the ${\dot{V}}_{E}/\dot{V}O_{2}$ relationship. Respiratory compensation point (RCP) was determined at the point of a non-linear increase in ${\dot{V}}_{E}/\dot{V}{\mathrm{CO}}_{2}$ and the first decrease in the expiratory fraction of CO_2_ [19]. Two independent investigators determined these thresholds; when the investigators disagreed, a third independent investigator was consulted. Typical error coefficient of variation is following: VO_2max_ = 3.0%, power = 1.9%, respiratory exchange ratio (RER) = 5.6%, time to exhaustion = 1.6%. In addition, test-retest typical error and coefficient of variation of lactate concentration ([La^-^]) measurements were 0.07 mmol/L and 5.2%.

#### Experimental testing sessions

Participants returned to the laboratory a minimum of one week after performing the $\dot{V}O_{2\max}$ test and familiarization to undertake the first of two randomly assigned exercise sessions (described below). After resting in the sitting position for ~5min they started the exercise. During both exercise sessions (resting, exercise, and recovery) gas exchange and HR data were collected continuously using the same automated breath-by-breath metabolic system described above. The HR and ${\dot{V}}_{E}$ data were averaged every 5 min of resting, exercise, and recovery to compare the LIE and LIE-BFR cardiorespiratory patterns. Blood samples to analyze [La^-^] were collected (earlobe) before each exercise session, 15 min during the exercise, immediately post- and 3, 5, and 7 min post-exercise and 15 min of recovery. Peak post-exercise [La^-^] was used for further analysis. Blood samples (25 μL) were immediately placed in microtubes containing 25 μL of 1g% sodium fluoride and then centrifuged at 3000 rpm for 5 min to separate the plasma before being aliquoted and frozen / stored at -80°C. Subsequently, plasma lactate concentration was measured using a spectrophotometer (ELx800, Biotek, Winooski, USA) using a commercial kit (Biotecnica, Varginha, Brazil).

The exercise protocols consisted of 30 min continuous cycling with a pedal frequency between 60 and 70 rpm. The LIE-BFR and LIE protocols were carried out at power output corresponding to 40% of $\dot{V}O_{2\max}$ (70 ± 9.8 W), determined at preliminary testing. The LIE-BFR protocol was performed with a cuff strapped over the thigh. Immediately before trials, subjects rested on a stretcher while the arterial occlusion pressure was measured. An 18-cm wide cuff was placed on the proximal portion of the thigh (inguinal fold region) and once in position, inflated until an absence of auditory blood pulse in the tibial artery was detected through auscultation with a vascular Doppler probe (DV-600; Marted, São Paulo, Brazil)[20]. Pressure was then slowly released until the first arterial pulse was detected, considered the systolic pressure in the tibial artery. Cuff pressure was set at 80% of the maximum tibial arterial pressure [20], the cuff was inflated throughout the exercise session and deflated immediately after exercise. The average of maximum occlusion pressure was 136±22 mmHg and average of 80% occlusion pressure was 109±18 mmHg.

#### Diet/Exercise control

Before each experimental trial, subjects were instructed to refrain from exercise training and vigorous physical activity, and alcohol and caffeine consumption for a minimum of 48h. In addition, subjects were asked to record dietary intake 24h before the first trial. In the posterior trials, subjects were asked to ingest a similar diet to the first trial.

#### Energy system contributions and energy expenditure

Blood lactate accumulation and oxygen uptake during and after exercise were used to estimate the anaerobic lactic metabolism, aerobic metabolism, and anaerobic alactic metabolism contributions, respectively [21]. The aerobic metabolism contribution was determined by the area under the curve of exercise $\dot{V}O_{2}$ and subtracting the baseline amount of $\dot{V}O_{2}$ corresponding to total exercise time (i.e., 30 min). To estimate the anaerobic alactic metabolism contribution, a mono-exponential model (Eq 1) was used to fit oxygen uptake recovery data (10 min after exercise) [21], and the amplitude multiplied by the time constant (Eq 2). To estimate the anaerobic lactic metabolism a value of 1 mmol∙l^-1^ [La^-^]_net_ was considered to be equivalent to 3 ml O_2_∙kg^-1^ body mass [22]. Total energy expenditure was calculated as the sum of the three energy systems. These estimates were performed using free software specifically developed to calculate energy system contributions (GEDAE-LaB, São Paulo, Brazil), available at http://www.gedaelab.org [21].

$$\dot{V}O_{2(t)}=\dot{V}O_{2baseline}+A_{1}[e_{1}^{-(t-td)/t_{1}}]$$ (1)

$$AL_{MET}=A_{1}∙\tau_{1}$$ (2)

Where $\dot{V}O_{2(t)}$ is the oxygen uptake at time t, $\dot{V}O_{2\mathrm{rest}}$ is the oxygen uptake at baseline, A is the amplitude, *td* is the time delay, *t* is the time constant, and _1_ denotes the fast component, respectively.

### Statistical analyses

Data normality and variance equality were assessed through the Shapiro-Wilk and Levene’s tests. The paired T-test was used to perform comparisons between conditions for study mean dependent variables: total energy expenditure, aerobic metabolism, anaerobic alactic metabolism, and anaerobic lactic metabolism. A two-way (*time* vs. *condition*) ANOVA for repeated measures was used to compare the [La^-^], HR and ${\dot{V}}_{E}$ responses. When a significant F value was found, the Bonferroni Post Hoc was performed to localize differences. Data are presented as Mean ± Standard Deviation (SD). The significance level was set at P ≤ 0.05. Statistical analysis was performed using SAS version 9.3 for Windows (SAS Institute Inc., Cary, NC, USA). In addition, the statistic power was calculated using G*Power 3.2.1 software, with a type I (α) error rate of 5%, sample size of 12, and the specific correlation and Cohen's effect size among the repeated measures of each main dependent variables [23].

## Results

### Energy system contributions and total energy expenditure

**Fig 1:**
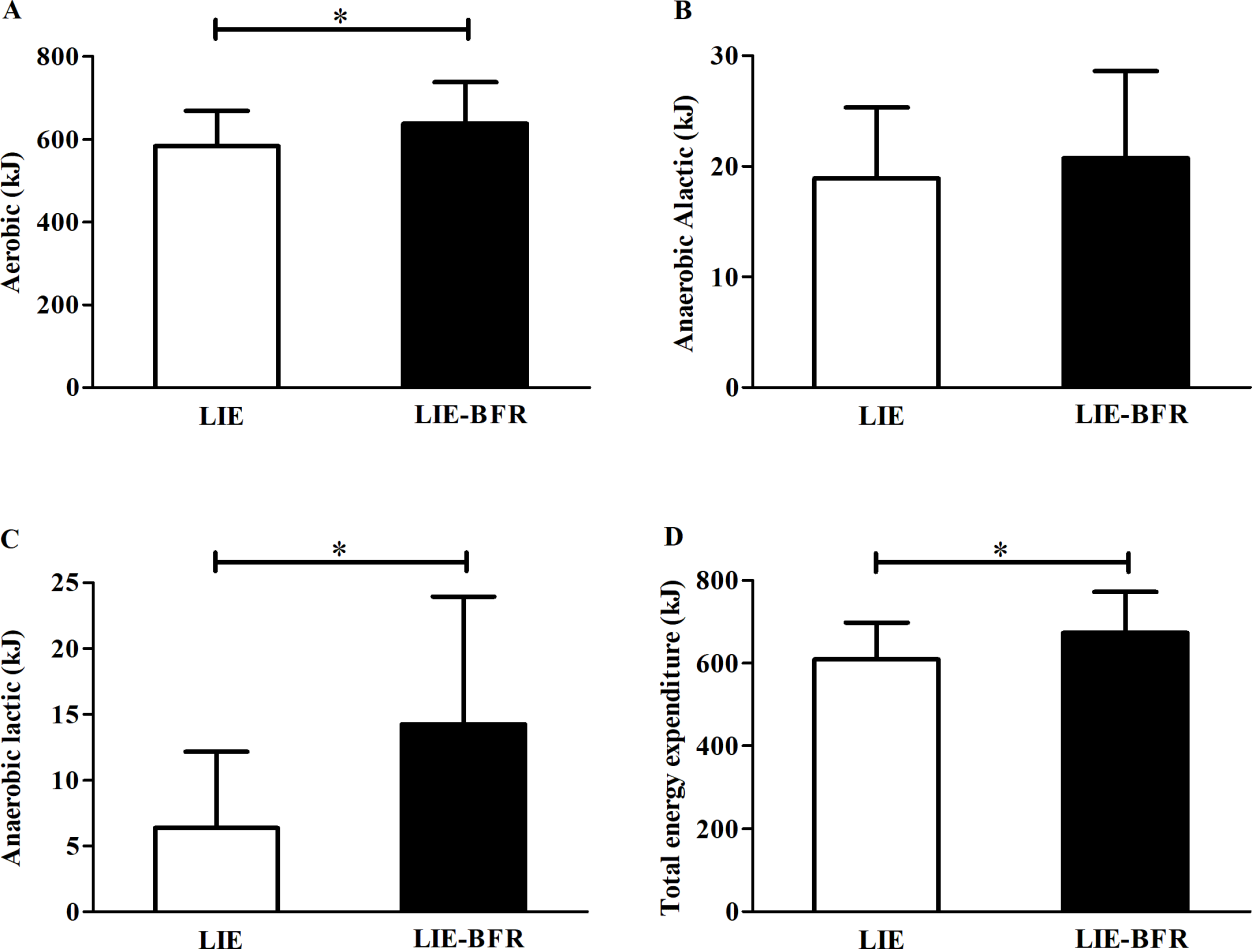
**Aerobic metabolism (A), anaerobic alactic metabolism (B), anaerobic lactic metabolism (C), and total energy expenditure (D) during low intensity endurance exercise (LIE) and low intensity endurance exercise with blood flow restriction (LIE-BFR).** Values are mean ± SD (n = 12). * Significant difference between groups (P ≤ 0.05).

Fig 1 presents the energy system contributions and total energy expenditure during exercise. There were significant increases in the aerobic metabolism (Fig 1A, P = 0.020; statistic power = 0.89), lactic metabolism (Fig 1C, P = 0.008; statistic power = 0.99), and total energy expenditure (Fig 1D, P = 0.008; statistic power = 0.96) with the LIE-BFR. No significant differences between conditions were found for the anaerobic alactic metabolism (Fig 1B, P = 0.582; statistic power = 0.34).

### Lactate concentration [LA-]

Table 2 shows plasma lactate concentration before, during, and after exercise. A significantly higher [La^-^] was found in the LIE-BFR compared with LIE at 15min (P = 0.008) during exercise, and peak post-exercise (P = 0.001). Plasma lactate concentration increased over time in the LIE-BFR (15min P = 0.004, peak post-exercise P = 0.04) and LIE (15min P = 0.003, peak post-exercise P = 0.03), returning to the pre-exercise levels after 15 min recovery for both protocols.

**Table 2:** Plasma lactate concentration responses to LIE and LIE-BFR.

Lactate (mmol·l^-1^)	LIE	LIE-BFR
Pre	1.32 ± 0.55	1.17 ± 0.44
15min exercise	2.14 ± 1.30	2.94 ± 1.10*
Peak post-exercise	1.97 ± 1.18	2.89 ± 1.49*
15min after	1.41 ± 0.67	1.55 ± 0.75

Values are presented as mean ± SD, n = 12. LIE: low intensity endurance exercise; LIE-BFR: low intensity endurance exercise with blood flow restriction.

* Significant difference between groups (P ≤ 0.05). Differences over time were omitted to improve clarity.

### Cardiorespiratory responses

**Fig 2:**
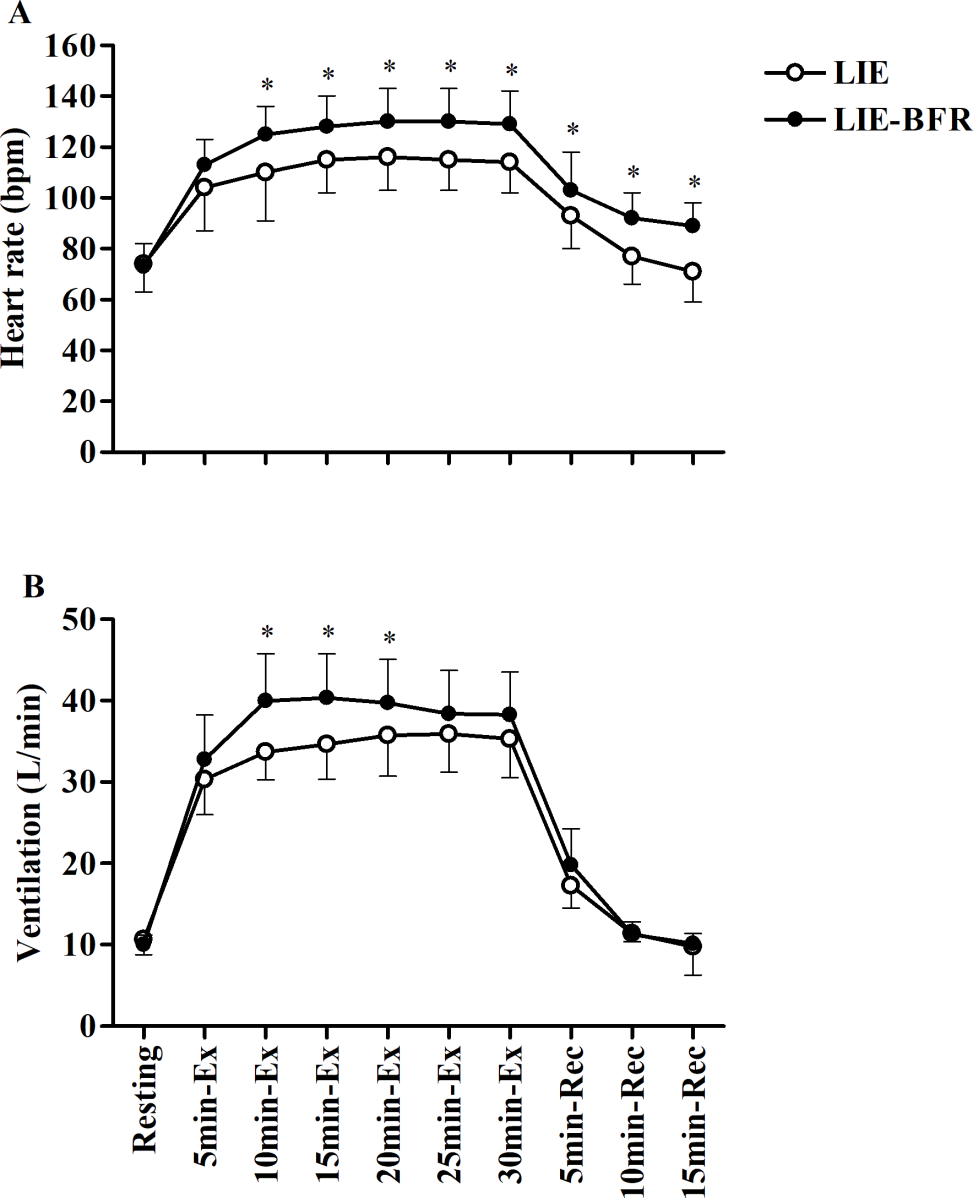
Heart rate and ventilation during and after low intensity endurance exercise (LIE) and low intensity endurance exercise with blood flow restriction (LIE-BFR). Ex: exercise; Rec: recovery. Values are mean ± SD (n = 12). * Significant difference between groups (P ≤ 0.05). Differences over time were omitted to improve clarity.

Fig 2 shows the heart rate (A) and ventilation (B) response to LIE and LIE-BFR. There were no significant differences between conditions for the HR and ${\dot{V}}_{E}$ at baseline. Heart rate was significantly higher in the LIE-BFR at 10 (P<0.0001), 15 (P = 0.0004), 20 (P = 0.0001), 25 (P<0.0001), and 30 min (P<0.0001) during exercise, and at 5 (P = 0.03), 10 (P<0.0001) and 15 min (P<0.0001) of recovery. Heart rate increased over time during exercise and recovery in both protocols (all P<0.0001), however, only in the LIE, returned to the pre-exercise level at 10 and 15min of recovery. Ventilation was significantly higher in the LIE-BFR at 10 (P<0.0001), 15 (P<0.0001), and 20 min (P<0.0001) during exercise. Ventilation increased over time during exercise and 5 min of recovery (all P<0.0001) returning to the pre-exercise levels after 10 min recovery for both protocols.

## Discussion

Low intensity exercise with blood flow restriction has been shown to promote increases in cardiorespiratory fitness [3, 4]. Accordingly, we hypothesized that local hypoxia induced by BFR would produce elevated muscular metabolic demand and could increase whole-body energy expenditure and cardiorespiratory responses. We report that low intensity endurance exercise performed with blood flow restriction increased the aerobic and anaerobic lactic metabolisms resulting in augmented total energy expenditure. Additionally, we found greater [La^-^], and HR and ${\dot{V}}_{E}$ responses in the LIE-BFR compared to LIE. Taken collectively, our findings suggest that cycling exercise undertaken with blood flow restriction is able to provoke additional perturbations to homeostasis necessary to induce improvements in $\dot{V}O_{2\max}$, which normally take place during moderate-vigorous intensity endurance exercise without blood flow restriction.

A growing body of evidence suggests exercise undertaken with blood flow restriction can enhance exercise adaptation [3, 4, 24, 25]. Some previous studies have reported that endurance walking/cycling exercise performed with blood flow restriction can increase cardiorespiratory fitness, although with smaller gains compared to high intensity endurance exercise alone [3, 4]. However, little is known about the mechanisms mediating these responses when low intensity endurance exercise is undertaken with BFR. As such, we recently reported that, despite great lactate response after LIE-BFR, there are no significant responses of genes and proteins related to mitochondrial biogenesis and angiogenesis after LIE-BFR [16]. In spite of the lack of significant results for these local markers we still believe that there is a link between local metabolic perturbations and cardiorespiratory adaptation induced by LIE-BFR.

It has been shown that BFR on leg muscles during exercise acutely changed cardiovascular responses compared to normal blood flow conditions [26, 27]. Although it is plausible to suppose that these changes are related to the effects of cuff-pressing the thigh musculature on changes of movement pattern, it was showed that decreases in locomotion economy with BFR were caused by the increased ventilation, which is likely matched to the rate of CO_2_ output [28]. Additionally, Ozaki et al., (2010) [27] showed that during cycle exercise at 20, 40, and 60% of $\dot{V}O_{2\max}$ with BFR can significantly increase HR and trend to increase $\dot{V}O_{2\max}$ while there was no significant change to the same exercise without BFR. Accordingly, Sakamaki-Sunaga et al., (2012) [29] compared cardiorespiratory and lactate responses to a graded walking test with and without BFR and showed an increased HR and $\dot{V}O_{2}$ at a given submaximal workload in BFR condition. Thus, it is suggested that the elevated cardiovascular response is due to the local hypoxia induced by BFR [5].

In fact, the pressure held by the cuff on the upper portion of each thigh induces an accumulation of blood into the legs with reduction in femoral venous return [30, 31]. Likewise, some evidence endorses the idea that there is a limited capacity for delivering O_2_ (e.g., reduced capacity of femoral arterial blood flow) to the exercising muscles and consequently decreased O_2_ availability [9, 32, 33]. As the O_2_ availability is decreased during exercise, the amount of energy provided by the anaerobic metabolism to maintain muscle contraction is increased [28, 34]. Confirming this rationale, we found herein that the aerobic and in particular anaerobic lactic metabolism was significantly higher during LIE-BFR compared to LIE (Fig 1A and 1C) which generates an increase in [La^-^] (Table 1). It is possible to suggest that the increase of [La^-^] identified in our study would be the result of the metabolite accumulation in the occluded vascular bed of the lower limbs, which would have restricted its circulation through the organism and, consequently its use as substrate by the muscles and other tissues [35]. However, we believe that this fact had less influence on the [La^-^] measurements, since with 15min of exercise, even with occluded blood flow, a higher [La^-^] was observed in earlobe samples collected from the LIE-BFR condition. This indicate that even with BFR the lactate circulated throughout the body and was probably used as a substrate as well as in exercise without BFR. In this way, it is important to note that even though it has been shown that the aerobic metabolism is the main energy system during exercise performed below $\dot{V}O_{2\max}$ intensity [36], it is plausible to consider that the anaerobic lactic metabolism is an important source of energy to sustain muscle contraction during LIE-BFR. Therefore, we suggest that the perturbation induced by BFR in arterial and venous leg blood flow increases the participation of the anaerobic lactic metabolism due to reduction in O_2_ availability, possibly resulting in the increase of HR and ${\dot{V}}_{E}$ in an attempt to adequately provide O_2_ to the exercising muscle.

By decreasing the availability of O_2_ (reduced arterial and venous leg blood) to the exercising muscles and retaining the need for energy production to maintain muscle contraction, it is also plausible to suppose that cycling with BFR might increase cardiorespiratory response by metaboreflex [37] and/or by decreased venous return [6, 29]. During BFR exercise the metabolic stress (e.g., [La^-^], Pi, pH) will be increased [38, 39] and stimulate metabolically sensitive group III and IV afferent nerve endings within the active muscle, eliciting a reflex increase in efferent sympathetic nerve activity and systemic arterial pressure, known as muscle metaboreflex [12, 40], a reflex that significantly contributes to the autonomic cardiorespiratory response to exercise, as well as, increasing ${\dot{V}}_{E}$ and HR [13, 41]. In fact, we have found that not only ${\dot{V}}_{E}$ was increased during exercise, but also the HR response during and after exercise was significantly higher in the LIE-BFR compared to LIE (Table 2), suggesting metaboreflex was activated and also cause a delay parasympathetic reactivation [42–44]. Another mechanism that potentially could contribute to increase HR response in this condition is the decreased venous return. Renzi et al., (2010) [6] investigated the effects of 2-minute treadmill walking at 2 miles/hour with 1-min interval either with or without BFR. They found that while exercise with BFR increase HR the venous return decrease, tanking in account that stroke volume is proportional to ventricular filling and thus the amount of blood returning to the heart by the venous vessels tree, it is possible to suggest that the HR increased as a compensatory maneuver to increase blood flow and O_2_ availability to working muscles. Taken together, it is suggested that metaboreflex and blood venous return are involved in HR response during and after exercise with BFR. Additionally, our data supports the idea that HR responses during and after exercise depends of on exercise intensity performed.

The normal blood flow to exercising muscle during exercise at 40% of peak workload seems not to elicit cardiovascular responses, while exercise at 60% of peak workload can induce an important pressor effect [37]. Although both our exercise protocols were performed with mild intensity exercise (40% of power output at $\dot{V}O_{2\max}$), it seems LIE-BFR induced a great combination between intensity and local hypoxia to increase ${\dot{V}}_{E}$ and HR, probably resulting from metaboreflex and reduced blood venous return. Altogether, we speculate that decreasing the availability of O_2_ that culminates in the high participation of the anaerobic metabolism and further in the aerobic metabolism during exercise, accompanied by increased HR and ${\dot{V}}_{E}$, might increase cardiorespiratory stimulus and thus improve $\dot{V}O_{2\max}$ after accumulated training sessions of LIE-BFR. Following this rationale, the link between metabolic perturbation and cardiorespiratory adaptation induced by LIE-BFR is the energy demand. Whilst this is an interesting hypothesis we suggest further chronic studies to confirm whether LIE-BFR can increase cardiorespiratory adaptation and studies testing different cuff pressure and/or exercise intensities that verify the contribution of increased anaerobic metabolism to consequent cardiorespiratory overload.

A second hypothesis emerges from this find, since even LIE-BFR is performed at low intensity, the present data shows that LIE-BFR can induce significant energy expenditure which could consequently induce weight loss [45]. Regarding practical terms, it is attractive to suspect that the LIE-BFR could be a promising exercise strategy for people that are not able to perform exercise with moderate/high intensity and need to cardiorespiratory fitness and lose weight as well as elderly and obese people. Accordingly, Karabulut and Garcia (2017) [46] showed that obese subjects cycling with BFR increased energy expenditure and cardiovascular stress. However, it is important to highlight that endurance exercise performed with BFR shows lower cardiovascular stress, measured by heart rate variability and hemodynamic responses, to low load with BFR (40% $\dot{V}O_{2\max}$) compared to high load without BFR (70% $\dot{V}O_{2\max}$) in elderly [47], suggesting the safety of this physical exercise method. It is relevant to note that the features of the present study (sample characteristics and experimental design) does not allow us to draw definitive conclusion concerning the importance of the LIE-BFR for special populations as elderly and obese subjects, or even reflect in additional benefits to a period of training. Thus, long term studies with different populations could be conducted to test these hypotheses.

In summary, this is the first study to investigate the contribution of the energy systems (aerobic, anaerobic alactic, and lactic) during LIE-BFR. Herein we show that low intensity endurance exercise performed with blood flow restriction increases anaerobic lactic and aerobic system contributions, total energy expenditure, and cardiorespiratory responses. Longer training programs incorporating endurance exercise with BFR that correlate measurements of the contributions of the energy systems with adaptation responses such as changes in $\dot{V}O_{2\max}$ and body composition will yield important information on the efficacy of this training method. If confirmed LIE-BFR training may become an important strategy to enhance training adaptation and improve health outcomes in populations that may be unable to perform prolonged/intense exercises, such as elderly and overweight/obese people.
